# Handling and Training of Wild Animals: Evidence and Ethics-Based Approaches and Best Practices in the Modern Zoo

**DOI:** 10.3390/ani13142247

**Published:** 2023-07-09

**Authors:** Sabrina Brando, Max Norman

**Affiliations:** AnimalConcepts, 03725 Teulada, Spain; info@animalconcepts.eu

**Keywords:** animal wellbeing, animal training, zoo animals, learning, positive reinforcement, human–animal relationship, capacity building, refinement, 3Rs

## Abstract

**Simple Summary:**

Over the past century, the field of animal care and management has changed in many ways. Changes to animal care strategies are a reflection of our increased knowledge and understanding of the capacity of animals to experience suffering and positive well-being states and a growing awareness of the need to respect all animals we work with. This includes the way animals are handled, trained, and interacted with in all contexts, and includes all taxa from the smallest invertebrates to the largest mammals. It is pertinent to review the methods of handling and training animals, including wild and exotic animals living in zoos and aquariums, on a regular basis with respect to current knowledge, understanding, and best practice.

**Abstract:**

There is an ethical responsibility to provide all animals living in human care with optimal and positive well-being. As animals living in zoos and aquariums frequently interact with their human caregivers as part of their daily care routines, it is both relevant and essential to consider the impact of these interactions on animal well-being. Allowing animals to have choice and control in multiple areas of their lives, such as by providing opportunities for them to voluntarily participate in their own care through, for example, positive reinforcement training, is an essential component of good animal well-being programs. This review aims to describe evidence-based approaches, ethics, and best practices in the handling and training of the many taxa held in zoos and aquariums worldwide, drawing from work in related animal care fields such as laboratories, farms, rescue, and sanctuaries. The importance of ongoing animal well-being assessments is discussed, with a particular focus on the need for continued review and refinement of processes and procedures pertaining to animal training and handling specifically. Review, enquiry, assessment, evaluation, and refinement will aim to dynamically support positive well-being for all animals.

## 1. Introduction

Animal welfare science and best practice in animal care should focus on the well-being of individuals, in respect of the fact that positive welfare is not something we give to animals but rather something they experience. Through a holistic approach, professional zoos and aquariums (henceforth zoos) should strive to promote optimal welfare for all animals in their care, utilizing a combination of modern science, best practice, and ethical frameworks, including compassion and empathy [1]. Being professional means having up-to-date theoretical and practical knowledge of animal welfare topics including but not strictly limited to learning, training, housing, environmental enrichment, and nutrition [2]. There is no lack of practical examples nor publications on the ways in which we can improve well-being for those animals living in human care, including in zoos, and it is our moral obligation to ensure that this theoretical knowledge and new best practices are integrated into our every day and systemic processes.

Despite significant advances in the care of captive wild animals and an increased understanding of the importance of individual experiences to overall well-being, suboptimal and outdated handling methods are still used for some species and individuals. For example, chasing small and more skittish animals into holding areas (pers. observation, Max Norman, 2021), or using physical restraint for animals that are easily grabbed or for individuals who are perceived as more aggressive. Flight and fear responses are often used for many taxa (earliest pers. observations Sabrina Brando, 1992) despite advances in animal training methods and the numerous known benefits of training animals to participate in their own care. With respect to growing knowledge as to the benefits of using training over coercive “traditional” methods, how animals are positively handled and trained should be at the core of any animal care and well-being program.

Russel and Burch [3] suggested the “3Rs” principles of Replacement, Reduction, and Refinement in the 1960s as an ethical foundation for using animals in research and, while the 3Rs were developed for animals in research specifically, the concepts proposed have broad potential applications when considering animals housed in other contexts including zoos and aquariums [4]. Although the principles can be interpreted in different ways [5], they are broadly described as follows [6]:

1. Replacement: The use of animals should be replaced by non-sentient animals or non-animal methods whenever possible without compromising other objectives, such as education, conservation, and research.
2. Reduction: The smallest possible number of animals to meet the objectives of the facility should be used; with the purpose of reducing collective animal harm.
3. Refinement: This principle entails that all procedures must be designed to minimize the pain and/or discomfort they cause to the animals, e.g., using anesthesia and analgesia, establishing humane endpoints to avoid suffering which cannot be relieved by other measures, and training animals with positive reinforcement.

Given the focus on improving animal well-being through careful consideration of what practices are necessary, the principles of the 3Rs are relevant to discuss and explore when considering how we can improve upon existing animal handling and training practices. Minimizing negative experiences and increasing positive experiences through refining animal handling practices aligns with other measures of animal well-being, such as the notion of creating ‘a life worth living’ for animals and meeting the criteria for good quality of life scores [7,8] In 1994, Mellor and Reid [9] developed the Five Domains Model for assessing animal welfare. This paper considered the importance of care inputs such as environment and nutrition in influencing their mental state; contemporary animal welfare frameworks over the decades since encompasses the multitudes of physical, behavioral and psychological aspects that are key in promoting positive welfare states [10]. Such modern frameworks also incorporate the necessarily subjective consideration of the animals’ emotions [11,12].

The aim of this review is to analyze and discuss past, present, and future trends in the handling and training of wild animals living in human and care. The literature concerning handling and training of animals in a variety of contexts will be brought together to form a thorough and holistic evaluation, informed where appropriate by the experience and expertise of the authors and others working directly with zoo and aquarium taxa. The ultimate goal is to synthesize a modern framework for the handling and training of animals living in zoos and aquariums that considers and incorporates the latest science, the needs and preferences of individuals, and overall promotes optimal animal well-being.

## 2. Animal Learning and Training

Animals are learning all the time, whether we are cognizant of it or not. Learning is described as the ongoing, dynamic, and individual process of acquiring new or modifying existing knowledge, behaviors, and preferences [7]. Training animals through positive reinforcement training (PRT) promotes the voluntary participation of animals in practices that are commonly regarded as negative, such as veterinary procedures or transport, through a process of encouraging behavioral change through rewarding animals for exhibiting desired behaviors. This is not at all a new concept; there are many publications on the topic, including explorations of reinforcer preferences and effectiveness [13,14]. However, while the use of PRT has greatly increased globally, it is not widely used as a standard practice but instead often seen as something that is done when there is time. This is particularly true for species beyond the larger, more commonly trained, and charismatic species such as elephants, great apes, and marine mammals [15]. Nonetheless, the widespread implementation of PRT has shifted common animal handling practices away from imposing coercive physical and chemical restraint practices and towards the willing, voluntary collaboration of the animals. Providing the animals with a degree of control increases the likelihood of handling being perceived as a positive experience while increasing the efficiency of procedures such as health checks and sample collection [16,17,18]. While the training of animals in zoos is a well-established practice, it is important to highlight that peer-reviewed articles concerning the handling and training of non-primate taxa in zoos are less common and much of the existing literature focuses on animals housed in research facilities. It is therefore pertinent to not only review sources from more commonly researched and domestic species but also from other sources such as the non-peer-reviewed “grey literature”, husbandry guidelines, and the first-hand experiences of those working in the field.

It should be recognized that learning occurs during all interactions between animals and their caregivers, both direct and indirect. For example, animals become familiar with indirect and informal actions, such as the daily routines of their caregivers and the clothes the veterinarian wears during medical examinations [19]. They are also cognizant of and remember the manner in which direct and formal animal care activities, such as feeding and training, are conducted and will learn from such experiences. As an example, during their daily routines care staff may always feed in certain areas and make it predictable for the animals where the food will be delivered, as well as the approximate time, which can be the cause of anticipatory and undesired behaviors [20,21,22]. While the focus of learning and training is often on formal training sessions, it is of utmost importance that those involved in the care of animals are attentive to the different processes and procedures happening around the animal on an informal basis as well. Informal training can take many different forms and occur through many different means. Behavioral and environmental changes may act as indicators that allow and provide animals information to predict upcoming events; for example, caretakers can wear different clothing when capturing animals or use sounds and other signals to communicate that a particular event is about to happen. Rimpley et al. [23] found that making certain husbandry events reliable and predictable through the introduction of a unique signal, such as knocking before entering their enclosure, had a significant positive impact in reducing the prevalence of anxiety-related behaviors in capuchins; in this example, the capuchins learned to associate the knocking with the door opening and a keeper entering. Such approaches are effective and easy to implement without any need for additional costs and are not time-consuming for caregivers. The absence of these signals will also serve as a communicative tool; when the signal is not given, the animals know not to expect the event to occur.

## 3. Choice and Control

The potential benefits of giving opportunities for animals to exert choice and control have been well described as long ago as the late 1980s [24,25] and have been extensively discussed in more recent reviews [26,27]. Several studies have established that having choices available, whether they are acted upon or not, is rewarding to animals [28,29] and thus the goal should be to allow animals far greater control over their lives with less dependence on caregivers [2,29,30,31,32]. This philosophy is particularly relevant in relation to training and handling. Communicating what is about to happen, for positive as well as possible negative events, gives animals the opportunity to decide what they want to do in response. Predictability and control have numerous benefits for animal well-being and have been subject to much recent exploration and discussion [14,33].

By focusing on what the individual animal wants and prefers, caregivers can plan and implement creative and empathetic training procedures. Different individual animals may have different preferences for food ingredients, for example, and thus find one type of reinforcer more rewarding than another [34]; meanwhile, other individuals may prefer non-food reinforcers, such as play, access to conspecifics, or toys [35,36]. The opportunities presented by training sessions can be extended by including not only options to choose reinforcers but also through the types of behaviors presented, who they train with—both other animals and human trainers—as well as location, and the option not to participate as discussed above.

Considering that animals are in their home environment on a near-constant basis and that there is limited staff for as many as 16 h a day [1,2] it is imperative to explore how learning and training can also aid in providing choice and control for animals when caregivers are not present. One of the most important aspects in animal care and well-being programs is the design of the environments the animals live in, including the main habitat as well as the back-of-house areas [1,37], and the options afforded to self-maintain. Despite only spending a limited amount of time with their caregivers, animals in human care are largely reliant on caregivers to meet all their needs and make choices for them. The implementation of operative technology such as touch screens, tokens, and motion sensors are just a selection of methods for creating environments where animals can make choices independently from humans with the aid of learning and training [1,2,37]. For example, animals can be trained to push a button or switch to activate systems such as heaters, light systems, or sprinklers [32,38,39,40] to provide additional control over their home environments.

## 4. Human–Animal Relationships

Human–animal relationships are defined as mutually beneficial and dynamic relationships between a human and animal participant, formed through repeated, consistent, and positive human–animal interactions [41]. Considering the human–animal relationship includes taking the perspective of the animal and how animals see those humans who are caring for them into account [42,43,44]. Animals have been demonstrated to recognize and form bonds with individual caregivers; for example, Davis and Gibson [42] documented the ability of a variety of research animals to discriminate between handlers based on the strength of their bond. It is highly likely animals of many taxa possess similar capabilities to distinguish between familiar and unfamiliar caregivers, as was documented by Martin and Melfi in African elephants, Rothschild’s giraffe, Brazilian tapir, and meerkats [45]. Consequently, animals who have experienced negative events with people, such as poor handling, undesirable changes in their environment, and invasive procedures may display avoidance and fear when interacting with specific or all humans in the future [46,47,48,49,50]. However, compared to animals housed in other contexts there is limited research concerning caregiver–animal interactions in zoos and aquariums.

The formation of relationships is beneficial for both caregivers and the animals in their care, reducing stress for the animals [51,52]. For instance, laboratory chimpanzees (*Pan troglodytes*) were more active and showed a decrease in abnormal behaviors when given an extra 10 min of contact with a familiar caretaker during the day [53]. Often, it is during daily animal management routines that caregivers and animals have their closest and most frequent interactions. Simple interactions, such as how caregivers approach the animals as well as the tone of voice they use when carrying out their daily activities, can have an impact on animals and how they perceive that caregiver. For example, gorillas (*Gorilla gorilla*) and chimpanzees displayed less self-directed behaviors during unstructured sessions with animal caregivers characterized by positive interactions in one study [54], which was consistent with the results of earlier work that demonstrated gorillas who had more training and play sessions with their caregivers displayed lower rates of abnormal behaviors and increased intraspecific play [55]. By contrast, negative or neutral interactions between animals and caregivers will not be conductive to a mutually beneficial relationship and these positive effects will not be seen.

These activities and bond-forming should start early in an animal’s life; Tuli et al. [56] reported that animals who are gently handled in the early phases of their life, as well as those who are habituated to a particular handler, show less stress related to handling in later life. Of course, provided interactions are positive, consistent, and repeated, positive relationships can be built with animals in all contexts. Livestock who are regularly pet and have caregivers who move slowly and are gentle with their approach have less fear of humans in the future [57]. Training to reduce fear can both foster the human–animal bond and reduce stress [58,59], thereby improving well-being; Bassett et al. [60] suggested that the increased human contact gained through training influenced a decrease self-scratching behavior, a sign of anxiety and fear, in marmosets (*Callithrix jacchus*). Overall, refinements in training methods that reduce fear and distress and increase cooperation between animals and their caregivers, with the animal’s experience as the focus, are key to promoting positive animal well-being [61,62].

## 5. Time

Time is often given as an obstacle or as a finite resource that must be invested into activities, such as spending time with the animals to build more positive relationships. Even when caretakers are on site they are only spending a limited amount of time with the animals, with much of their schedules taken up with meetings, preparing food, maintenance, and cleaning habitats for the often large numbers of animals they are responsible for the care of [2]. Given the often incorrect perception that PRT is time-consuming, other methods of training are often relied on instead whether caregivers are cognizant of this fact or not. For example, animals may be reinforced through positive punishment (e.g., adding the squeeze in the cage when the animal does not come forward for voluntary blood sampling), negative punishment (e.g., removing or reducing access to food or water), and negative reinforcement (e.g., removal of an undesired stimulus such as a catching net when the animal moves forward towards the desired location). These examples refer specifically to forms of operant conditioning before even considering forms of classical conditioning that may inadvertently be used. The animals are learning, but without specific time invested into the use of intrinsic motivation through positive reinforcers the outcome is likely not a positive one from the perspective of the animal [63]. While aversive methods may save time, they are in conflict with the aims of achieving excellence in positive animal well-being and empowering animals through best practice. Meeting these goals requires taking an in-depth, honest, and holistic look at the methods of handling used and the compromises that are being made to save time or recognizing when there is a lack of official and contemporary animal training program as a whole.

Reflecting on current best practices includes acknowledging the need to spend time not only on PRT, but also on writing up detailed training plans, considering ethical processes [64], evaluating risk assessments [65], and monitoring the outcomes of training. Putting in the time to build a rapport with the animal outside of training could help overcome many challenges common in animal care, such as negative responses from animals to veterinarians as well as sometimes towards the care staff themselves. As a practical example, veterinarians should be included in animal training and habituation programs acknowledging that, in most cases, animals will otherwise only encounter veterinarians during unpleasant circumstances. If veterinarians or other veterinary professionals are only present when animals are unwell, administering treatments that may be unpleasant, or touching animals in ways that they are uncomfortable or unfamiliar with, it is likely that the repeated negative human–animal interactions will lead to less favorable responses towards veterinarians in the future. Such outcomes may be avoided by giving veterinarians the time and opportunities to get involved with daily animal care including feeding and enrichment to allow animals to become accustomed to their presence as one that is enjoyable and pleasant. Ensuring that the time is available for these positive interactions to occur should be seen as fundamental to animal well-being.

## 6. Species

Attention to learning and training is relevant in the handling of many animals housed in zoos, including primates, large carnivores such as canids and big cats, birds, elephants, and marine mammals. Other less-considered animals such as hoofstock, large and small, as well as fish, reptiles, and small mammals can also be trained to participate in their care [66,67]. While modern animal care must also be informed by the latest science and understanding and best practice, many species, particularly non-mammalian taxa, have not received extensive attention in the literature surrounding PRT [68]. Nonetheless a lack of current evidence base should not preclude zoos and aquariums experimenting with PRT methods for all species. In general, future research would benefit from an increased focus on the species-specific impacts of PRT over other forms of traditional handling methods [69]. In the following section, we will go into more detail on specific handling methods, theories, and best practices relevant to major taxa held in zoos and aquariums. While the list of species that may be held is exhaustive and therefore not possible to discuss in entirety, this discussion is broadly applicable to most species that one may encounter in a zoo or aquarium context.

### 6.1. Non-Human Primates 

Non-human primates broadly include all species of great and lesser apes, old world and new world monkeys, and prosimians including lemurs and galagos. Handling methods will vary depending on the species; for example, while many new world monkeys are small and largely safe to interact with in the same space [70,71,72], legislative and safety requirements are often in place to prevent direct contact between humans and great apes such as placing two levels of barrier between caregivers and animals [73,74,75].

Direct handling and physical restraint using tools such as nets may be used for primates which are small and safe for human caregivers to share a space with, such as many new world primates [76]. For larger primates, it is not practical nor safe to utilize such methods; under modern zoo legislation and health and safety guidelines, such as the Zoo Licensing Act (1981) in the United Kingdom, caregivers are often not able to enter the same spaces due to the risk of injury or disease transmission in either direction. Consequently, many facilities utilize forms of chemical restraint, such as general anesthesia, for the purposes of veterinary examination where appropriate. Nonetheless, catching and restraining primates through these methods is a highly stressful and negative experience for the animals when they are not trained to expect it; as such, modern best practice should seek to replace or refine them with PRT methods wherever appropriate and possible [3,77]. Indeed, primates are one of the most commonly trained species in zoos with many examples of successful training programs [78,79,80,81,82].

Primates can be reliably trained to cooperate in providing samples such as urine, fecal, and blood samples. Such training reduces the need to restrain the animal physically or chemically for sample collection, as well as ensuring urine samples are not contaminated and are in sufficient amounts for reliable health monitoring and research. For example, laboratory-housed common marmosets were target trained for purposes of home cage weighing and taught to provide urine samples using PRT [62]. An increase in sample quality was also noted by Smith et al. [83], whose training of three species of callitrichid primates using PRT increased the quantity and speed of urine collection, as well as the proportion of animals which participated in collection. In another example, macaques were trained using PRT to present a leg during blood collection—a procedure which traditionally involves stressful restraints [84]. Training reduced the cortisol responses and defensive behaviors which often accompanied traditional restraint, suggesting an improvement in well-being. Similar methods have been used for macaques and chimpanzees [85]. The practical benefits of training zoo-housed primates to their care and well-being are extensive. For example, the condition of a terminally ill douc langur (*Pygathrix nemaeus*) could be monitored using hand injections [86]; in another case study, a golden-bellied mangabey (*Cercocebus chrysogaster*) was trained to co-operate in its own healthcare for diabetes management [87]. Training can also be used to fit devices for medical monitoring, including for overnight care; for example, slow loris (*Nycticebus bengalensis*) in one zoo were fit with accelerometers using PRT techniques [88].

Movement of animals between different areas of a habitat or for transport is another area where PRT has been broadly beneficial to the care of primates [89,90]. The ability to voluntarily move animals allows caregivers to safely enter enclosures, close off areas for cleaning, or transport animals for veterinary examinations. Squirrel monkeys *(Saimiri sciureus*)*,* in one example, were trained through PRT with a clicker as a conditioned reinforcer to voluntarily participate in transport [91]; a majority of the squirrel monkeys in the study learned all behaviors within two months, which enabled more efficient handling of the group. In one group of 30 sooty mangabeys (*Cercocebus atys*), PRT was utilized to train animals to move from one side of their habitat to the other [90]. Though more challenging than training singly housed primates, the training was still successful in gaining cooperation from most of the individuals. Training can also be used to move individual animals away from the social group voluntarily [16], a situation that is usually very stressful for social species [82].

Such methods are broadly applicable to a variety of different species; 17 different species of New World Primate at the Bronx Zoo, for example, were trained using PRT to cooperate in husbandry procedures such as weighing, syringe feeding, and crate training [80]. However, species-level differences should be noted, such as some species or individuals being more nervous or deliberate around humans [92] and requiring more time to become accustomed to the training compared to those who may be more confident [92,93,94,95,96]. Training can help adjust animals to the presence of humans without impacting their other behaviors through a process of habituation [97]. More attention should be given to these species-level differences in training responses [98] in order to refine the process at an individual level. For example, while species such as lemurs, lorises, and galagos are commonly trained in zoos, there is little empirical research available to document the impacts of training on these species compared to more often studied taxa such as macaques and chimpanzees [81].

### 6.2. Carnivores

Large carnivores compromise a great many diverse species including big cats such as lions (*Panthera leo*) and tigers *(Panthera tigris*)*,* canids such as wolves (Canis *spp*.) and wild dogs *(Lycaon pictus*)*,* hyena *(Crocuta crocuta* and *Hyaena hyaena*)*,* and ursids. However, the recommendations and discussion put forward in this section may also be applicable to other carnivores often housed in protected contact situations including wolverines (*Gulo gulo*) and smaller wild cats such as servals (*Leptailurus serval*) and caracals *(Caracal caracal*). Broadly speaking, these discussions are also relevant to some marine mammals such as polar bears (*Ursus maritimus*) and large pinnipeds such as Steller sea lions (*Eumetopias jubatus***)**, who are also often managed in protected contact.

Depending on the species in question, safety guidelines for large carnivores commonly stipulate that direct contact with animals must be avoided with the necessitation of at least one barrier between human caregiver and animal [99,100,101]. Such protected conditions can make certain necessary husbandry procedures more challenging; for example, when animals must be shifted into different areas of the enclosure for caregivers to safely enter, clean, and reprovision their habitats. For both the former as well as procedures such as medical examinations and blood draws, husbandry training has been a successful approach for many of these species. While it has been demonstrated that animals may be slower to respond and more likely to refuse responses when housed and trained in protected contact systems [102], this effect is, most likely, a demonstration of improved well-being with increased choice and control rather than any failing of the part of the trainer and could be seen as a positive outcome.

Using a common domestic model as a foundation for training and handling large carnivores proves difficult. The closest approximation would be domestic dogs; however, in many cases, dogs do not require the same safety precautions and are often managed with direct contact which is far different from the handling of similar species in zoos such as large canids. Nonetheless, findings from dog and cat cognition may be more broadly useful for understanding the capabilities of large carnivores and their capacity to learn. Additionally, the negative effects of using aversive training methods in dogs and cats are well documented [103] and can be transferred to our understanding of training methods in large carnivores. In studies of dogs, confrontational and punishment-based training methods are associated with aggressive responses [104,105] and the same is likely to be true of other species. There is no evidence to suggest that punishment-based methods are more effective than positive reinforcement for the training of any species [104] and, as such, the use of aversive training methods poses an unnecessary risk to animal welfare and may additionally put human caregivers at risk. Coercive and forceful negative interactions, including between large carnivores and human caregivers, must be avoided in all cases.

Positive reinforcement has a long history of use with dogs and, consequently, there are many resources available which discuss the implementation of PRT methods for canines in further detail [106,107]. PRT has been used to great effect to aid with common care procedures in domestic small animal practice, such as in the transport of animals to veterinary practices [108] and such practices can be readily applied to zoo and exotic species. In the zoo context, PRT has been demonstrated to have positive impacts in reducing stereotypic behaviors in African wild dogs [109] and in hyenas [110]. Use of PRT for co-operation in transport procedures in one study had a positive effect on decreasing stress hormones present in feces in fishing cats (*Prionailurus viverrinus*), indicating reduced stress associated with transfers and introductions as a result of training [111]. PRT has been used to profound positive effect in protected contact scenarios with large carnivores and, in surveys of caregiver perceptions, is seen as the safest form of handling with the greatest benefits for both animal welfare and caregiver–animal relationships [112].

PRT allows for animals to be moved into different areas of their habitats using recall and for veterinary procedures to be carried out without need for chemical or physical restraint. Introducing animals gradually to touch through the barrier can improve their comfort with various husbandry procedures, such as touching body parts commonly used for blood draws, inspecting the ears, or touching their feet [113,114,115]. It is also possible to train animals to present body parts that would not otherwise be able to be examined without physically touching the animal. In one example with lions and tigers in a Japanese zoo, big cats were trained with PRT to lie down on a designated station and were habituated to their tail being gently hooked through a specialized gap in the barrier for blood collection [116]. Such strategies allow for samples to be collected without the need for chemical restraint or for significant enclosure design modifications.

The handling and training of large carnivores must be carried out with respect to other aspects of their management with consideration given to, for example, feeding styles and food presentation. Many carnivores are fed on fixed time intervals including “fast days”, also known as starve days in some parts of the world; for example, feeding animals every two or three days, or designating one day a week as a day for not feeding [117]. Such fast days can have negative impacts on behavior, for example, one study demonstrated that tigers paced more frequently on fasting days compared to days when they were fed [118]. More naturalistic feeding presentations, such as giving animals larger feeds including whole carcasses, may reduce these effects and improve welfare [119] and can be combined with successful animal training programs.

### 6.3. Small Mammals

When discussing small mammals, we speak about all mammals that are not kept in protected contact situations that are theoretically possible to directly handle and maneuver. Examples include meerkats (*Suricata suricatta*) and other mongoose in the family Herpestidae, small canids such as bush dogs (*Speothos venaticus*) and foxes (*Vulpes* spp.), raccoons (*Procyon lotor*) and raccoon dogs (*Nyctereutes procyonoides*) as well as the many diverse species of rodent, lagomorphs, small marsupials, and other species. While all species considered under the umbrella of “small mammals” are possible to directly handle, this is often not the best or safest course of action for the animal or for the human caregiver [120,121].

Small rodents such as mice and rats are easily handled and restrained for most procedures, and so instead of training them to cooperate in their care we often rely on more forceful and coercive handling methods including the use of nets and snares [122]. The same is true for many other species of small mammals. For those individuals that may react to handling with aggression, methods such as gloves or towels may be used to protect the human caregiver while the animal is physically restrained [120,121]. Such methods do not consider the perspective of the animal and how repeated negative experiences with being handled roughly and without the opportunity to exert agency over their care can lead to poor human–animal relationships [123,124,125], and thus increased likelihood of being fearful and aversive towards humans and handling in that moment and in the future. As an example, the traditional handling method for small rodents involves lifting from the tail, which is acknowledged to be aversive and a cause of stress and anxiety [126].

Gentle handling, especially of species known to be skittish or nervous around humans by nature—particularly prey species—should always be the priority when developing best practice guidelines for the handling of all animals. Non-aversive handling techniques such as the use of a tunnel, asking the animal to step on the hand and cupping the animal on the open hand can be used to reduce the stress associated with handling. For example, tunnel handling was demonstrated to improve the performance of laboratory mice in behavioral tests in one study [127] and thus such gentle handling strategies can be demonstrated to lead to improved welfare over other methods. Additionally, mice appear to benefit from being handled gently with more freedom to move across the palm [67]. The habitat design of small animals should also be a consideration, particularly for those prey species who may be more skittish. Providing access to hiding places or other retreats during training may seem counter-intuitive, but for animals who might want to hide it provides a safe place to go if they want to and gives a sense of perceived control [29]. In recent years, both authors have attended events and training programs which highlighted formal training and handling with small mammals which focused on a positive human–animal relationship with a gentle and voluntary handling style; however, peer-reviewed publications are much needed to provide evidence and descriptions of alternatives to standard operating procedures.

PRT can also be utilized to reduce the reliance on directly handling animals. Allowing animals increased agency through PRT promotes safe, positive interactions between all species of small mammal and their caregivers and may turn normally aversive procedures into enriching experiences. For example, meerkats at London Zoo were trained to voluntarily enter individual carrier crates so it could be possible to separate animals for transporting individuals to vets or for other purposes. Separation is a typically a negative and stressful experience for social animals [82]; however, by using PRT the meerkats could willingly and calmly choose to enter individual crates without the need for forceful handling. These methods have been successfully used for many mustelids and related species [120,121]. The Copenhagen Zoo has similarly trained guinea pigs (*Cavia porcellus*) involved in visitor programs to choose whether they will voluntarily enter a crate for transportation. The animals are also not handled by hand but instead enter a hammock which allows them to be comfortably moved between visitors. All of these actions increase opportunities for choice and control for animals involved in various programs.

Despite larger and more charismatic mammals often receiving extensive training, smaller mammals are less often trained. A survey of Japanese zoos found only a little over half of interviewed zoos conducted daily training with their red pandas [128]. Similar results have been found in surveys of other species and in other regions; a survey of wolverines (*Gulo gulo*) in Europe, for example, found that only 57% of respondents trained their animals [129]. Training for all animals should be considered, including making the time for such interactions to occur on a daily, weekly, and monthly basis. Increased attention to more frequent training for all animals is essential and has many benefits for animal well-being; more frequent training has been connected to increased reproductive success [130] and improved human–animal relationships [131].

### 6.4. Hoofstock

Hoofstock refers to all ruminants, including bovids, cervids, ovids, and extant giraffes including all subspecies of giraffe (*Giraffa camelopardis*) and the okapi (*Okapia johnstoni)*, as well as all members of the horse family (*Equus* spp.). The term hoofstock is also used in reference to other monogastric herbivores besides equids such as pigs, including wild pigs such as the warthog (*Phacochoerus africanus*), extant species of elephant, and all species of rhinoceros.

For smaller, more nervous hoofstock species such as sheep or small antelope, utilization of flight distance as a form of positive punishment to move animals away from a particular location through the addition of an undesired stimulus, such as a caregiver entering the designated space, is common. While this method does indeed work, the functionality of a method does not make it ethical when positive reinforcement can achieve the same results; for example, by calling the animals over toward the care staff for a food or other reward. For flight distance to work effectively, the caregiver must paradoxically go against ideas of positive human–animal interactions, as animals who are habituated and used to humans being a positive presence have shorter flight distances than animals who have poorer relationships with handlers [132,133]. In fact, evidence suggests that giving animals space when they approach caregivers may encourage more positive human–animal relationships where animals feel safe to approach caregivers and other humans in the future [134]. As an example, petting zoo sheep at Frank Buck Zoo were trained to be less fearful of humans using a negative reinforcement strategy wherein trainers would remove themselves when the sheep approached; all sheep later became habituated to human presence and could be trained with PRT once they were comfortable to eat around and take food from their caregivers [134]. Such strategies may be beneficial when, such as in the preceding example, animals are too skittish to eat around people and it may not be possible to reinforce them through normal methods. The context of reinforcement is essential to consider; while a negative reinforcement strategy is not easily avoidable when the reinforcer being removed is something ubiquitous to living in human care, such as the presence of humans in general, this type of negative reinforcement is often the starting point for animals who are not accustomed to people. Professional animal training programs will allow for a rapid transition from negative reinforcement or punishing methods to PRT as the priority strategy.

Unfortunately, PRT in hoofstock species is not as well studied as in other zoo taxa—potentially due to the extensive use of the traditional physical and aversive strategies that are commonly utilized for domestic livestock species used in farms. Domestic hoofstock are often managed using direct methods, often by hand or with specialized equipment, and many of these methods are variably used for zoo and exotic species as well [135]. For example, domestic sheep are commonly physically restrained for procedures such as shearing, while horses are commonly tied to posts while their hooves are trimmed or cleaned. Headcollars and halters are often used for various hoofstock species, such as equids, caprids, and camelids, particularly when moving them between different areas of their habitat or to restrain them in place for certain health and other care procedures. The management of hoofstock using welfare-conscious and positive handling methods has received limited attention in the literature, despite these animals frequently being subject to aversive physical methods to achieve compliance despite an understanding that these methods create fear from the animal’s perspective and put human caregivers at risk of injury [136].

In the past, remote delivery systems such as darts for vaccinations and sedatives were commonly used for the management and handling of larger hoofstock for veterinary procedures [137]. Many zoos and safari parks, particularly those with large semi free-ranging populations, still practice these methods. Remote delivery is, however, imprecise, and comes with the risk of soft tissue damage to the animal and in worst cases fractures and even mortality from either the dart itself or from falls upon sedation [138,139]. Furthermore, the equipment required to keep such large animals unconscious for veterinary procedures is expensive and extensive and often not practical for small interventions such as vaccinations, blood draws, and minor physical health checks. Wherever PRT can be used to train hoofstock to be receptive and voluntary participants in these procedures, risks to animal health and well-being are minimized with no compromise to the human–animal relationship. For example, crate-trained antelope and bison who co-operate with restraint for veterinary and other procedures have cortisol levels comparable to baseline in resting cattle [140,141], highlighting crate-training as a handling method that is non-stressful for the animals and thus at the very least a neutral if not positive experience. In one case, a pygmy hippo (*Choeropsis liberiensis*) was trained to receive daily dental care, a procedure that would otherwise require regular and invasive sedation for more intrusive trimming of the tusks. In another case, nyala (*Tragelaphus angasi*) were habituated to a crate for blood draws and demonstrated that PRT could be used to collect samples that were not confounded by stress [142]. Many other case studies demonstrate the benefits of PRT for enhancing animal well-being during husbandry practices in ungulates [143,144,145].

Wild swine species may be particularly receptive to PRT methods owing to similarities with their domestic counterparts. Equipped with excellent memories and quicker to learn cognitive tasks than dogs [146], domestic pigs easily learn to participate voluntarily in their daily care and with the use of positive handling techniques stress can be minimized [147,148,149] and it is not a stretch to assume that this can be generalized to species of wild pig as well. As long ago as the 1980s, Grandin [147] recommended training pigs for restraint with food rewards for desired behavior; such methods are utilized today in the training of wild pig species in zoos, for example, in one case red river hogs (*Potamochoerus porcus*) were trained to enter a cage for desensitization to vaccinations and health checks using a food reward. In another example, pigs used for biomedical research were trained to cooperate with a non-invasive biophysical skin measurement by associating the skin analysis with feeding time and positively reinforcing cooperation with exercise time outside of the home pen [149]. On the other hand, it has been noted that pigs can require more training to overcome past negative handling experiences when compared with other species [148]; the capability of animals to remember past experiences and react accordingly must thus always be considered in all interactions, both formal and informal.

### 6.5. Birds 

Birds have a long history of handling by humans in various contexts. Multiple species of bird, particularly pigeons (*Columba spp*.), parrots, and some corvids feature prominently in scientific literature, and humans share an extensive history with the many species of raptor involved in falconry and even warfare [150]. However, much of the literature focuses on reinforcement as a tool for learning and behavior modification and does not primarily discuss the implications of PRT for improving bird welfare; furthermore, the much wider range of bird species held in zoos including flamingos, pelicans, penguins, and more are not nearly as extensively discussed nor studied in the literature.

The historical, standard, and traditional method for handling laboratory pigeons involves restraining the bird upside-down in a cup for weighing [58]. Other aversive methods are often used for the handling of smaller and skittish birds such as relying on flight distance to move animals or physically cornering and grabbing animals for health checks [151]. Such standards are outdated, do not provide the birds with opportunities for choice and control, and involve handling methods that are stressful for the animal, and are not conducive to positive human–animal relationships [152]. While a traditional method may “work” for handling an animal and gaining the results that the caregivers need, they are in dire need of review to reflect how birds can be handled with respect to their perspective and their welfare and considering their intelligence and capacity to be trained effectively for husbandry procedures.

It is generally agreed upon that desensitization and positive reinforcement may reduce handling stress in laboratory birds [153], for example, adult laboratory macaws learned several behaviors related to voluntary participation in veterinary procedures such as stepping onto a scale or allowing a syringe to be pressed to the pectoral area [154]. Outside of the laboratory, managers at Edinburgh Zoo successfully utilized PRT in their husbandry training of Marabou stork (*Leptoptilos crumeniferus*); the process reduced aggression and stress during movement and health-checks [155]. Despite advances in our understanding of bird cognition and of the benefits of PRT in zoo animals, the implementation of positive and best-practice PRT for husbandry training in birds also lags behind developments for other taxa held in zoos and often predominantly focuses on outdated falconry training practices and relies heavily on poorly practiced weight management [156]. Weight management, by definition, is a good practice to monitor and maintain healthy weights for animals in human care. However, the overall lack of PRT and refinement practices in the training of raptors, apart from exceptions in various parks around the world, is a cause for concern and indicates a need for further research. The lack of development is made evident through the scarcity of literature concerning the training of birds specifically housed in zoos; for example, despite many species of penguin being commonly held in zoos and aquariums worldwide and practical examples being available in non-peer-reviewed media such as blogs, videos, and at conferences, there are limited available empirical studies concerning the welfare benefits of PRT for these animals.

A potential reason why bird training research and updated best practice lags behind that of other species held in zoos is that, owing to the long history of training birds for entertainment or other human uses, bird training is seen as more complex or more time-consuming and difficult when compared to other species. This is despite no evidence that this is the case. Indeed, many birds in zoos are trained for free-flying demonstrations given to zoo visitors and yet less often specifically for husbandry and management procedures, while birds held in exhibits on display may receive little or no training at all [151]. In the past, much of this free-flying training for bird shows surrounded inappropriate weight management under the assumption that hungry birds would be more motivated to perform desired behaviors during training; such presumptions are now seen as outdated, and bird training is evolving in many places with a focus on positive caregiver–animal relationships and PRT. Falconry and its related management methods have a long and extensive history; in-depth discussion of the management of birds of prey held in this manner is outside of the scope of this paper but has been subject to review [157,158]. Nonetheless, PRT has been demonstrated to have positive benefits for birds utilized for falconry; for example, in training animals to be desensitized to handling equipment such as gloves and to procedures such as having their feet touched and manipulated [159]. These benefits should be seen as broadly applicable to all species of bird whether they are managed as part of a free-flight or falconry program or in enclosed display aviaries.

### 6.6. Reptiles

Non-avian reptiles comprise a great many species including chelonians, crocodilians, lizards, snakes, and the lizard-like tuatara (*Sphenodon punctatus*). Handling methods of reptiles vary depending on the safety of working with the species; for example, different methods will be utilized when handling a non-venomous snake versus a venomous species, and while some species are relatively safe to handle others may pose too large a safety hazard for caregivers to share a space with.

It is often recommended that reptile management often takes a more “hands-off” approach that stipulates animals are only handled when it is necessary to do so. When the capture of a reptile is required, this is typically done by hand or using specialized capture nets [160]. Bite-proof gloves are recommended for handling some species which have been known to bite the hands or forearms of handlers [161,162]. In the case of snakes, specialized snake hooks are often used to move large and/or venomous snakes between enclosures, for transport, or for procedures [163]. As with mammals and birds, capture and handling through physical and direct methods have been demonstrated to be stressful for reptiles [164], leading to increases in corticosterone [164,165] and body temperature indicative of stress [166]. However, as reptile stress and welfare are less commonly studied than for mammals and is considered difficult to measure [167], reptiles are underrepresented in the literature of welfare responses to handling. Indeed, it is only in the last few decades that there has been an increased focus on training reptiles as an alternative to traditional handling methods, and even with perceptions of reptile cognition changing there are still limited studies demonstrating the effectiveness of PRT in improving welfare for reptiles. Nonetheless, it is likely that training reptiles for more effective handling with PRT has similar if not the same benefits for reducing stress and increasing well-being as seen in other taxa.

There is a misconception that reptiles do not learn or are not as cognitively complex as the more commonly trained mammals or birds, however, reptiles have been demonstrated to show a great deal of cognitive complexity [168]. They are, therefore, just as capable of learning and problem-solving; they can, for example, be taught how to solve simple puzzles to access food rewards [169] and trained for husbandry procedures [170,171,172,173]. In one study of adult Aldabra tortoises (*Geochelone gigantea*), the subjects were trained using a clicker and a food reward to stretch their necks and hold the position for blood draws [171]. Another case study presented a training procedure utilizing targets as a bridge for reinforcing shifting behavior in false water cobras (*Hydrodynastes gigas*) [173].

Not sharing a space with an animal that is liable to pose a safety risk or other hazard to the caregiver or itself should always be considered best practice and alternatives must be explored, such as the use of PRT, to facilitate husbandry that otherwise would require the animal to be restrained or sedated. As an example, a Nile crocodile (*Crocodylus niloticus*) was station-trained using PRT so that caregivers could obtain weight and blood draws without restraining or sedating the animal [170]. In another example, a gharial (*Gavialis gangeticus*) was trained to voluntarily enter a crate for transport [172]. While using food as bait can be problematic, such as when an animal must fast for a veterinary procedure, it is possible to train reptiles with targets to eventually phase out the need for bait.

### 6.7. Fishes 

Fish held in zoos and aquariums comprise a great many species, including both bony and cartilaginous fishes living in all manner of marine, freshwater, and brackish environments. Management and handling of smaller fish typically involve physical capture with nets, cups, tubes, or other containers, or indeed in the case of rays or other flatfishes, direct physical restraint may be used where it is easy to hold them down on the bottom of the pool by hand. There are few documented examples in the peer-reviewed literature of the use of positive reinforcement to train fish, and most of the literature that does exist focuses on training fish for research purposes as opposed to training husbandry behaviors. A positive reinforcement method was used to measure the auditory sensitivity of goldfish (*Carassius auratus*) and, while the training did not involve husbandry, it did utilize a training paradigm which involved no use of physical punishment as was common for similar experiments that came before it [174]. Fish are additionally demonstrated to respond to common operant conditioning procedures used in other species, such as target training; as an example, in a study on free-living *Tripterygion tripteronotum,* fish were target trained within 10 days using food rewards [175].

While there are examples of using PRT for husbandry purposes with fish, including many great videos on YouTube and informal reports in the non-peer-reviewed grey literature, and examples of the positive influence PRT has on fish welfare, much of the evidence that currently exists has not been published in peer-reviewed journals [67]. This is despite some larger species having described training procedures [176]. Future research directions should explore how informal reports could be empirically reviewed and shared to a greater extent, such as through greater collaboration between aquariums and research institutions such as universities and by encouraging animal caregivers to record and publish their work. As an example, in a study published by an aquarist in the USA, PRT was used on zebra sharks (*Stegostoma fasciatum*) in the Downtown Aquarium to reduce negative associations with veterinary procedures; all zebra sharks learned to cooperate with typically stressful stimuli, with the hope that this would reduce stress during health checks and examinations [177]. Training of fish for husbandry and veterinary procedures is nonetheless still relatively rare, potentially owing to a lack of appreciation and understanding of the ability of fish large and small to experience suffering and stress from aversive handling and management.

Nonetheless, PRT may be used to make the husbandry of fish simpler for aquarists, who frequently may be diving within the same tank as animals to perform husbandry tasks such as feeding, cleaning, and health checks. Problem behaviors can develop in species such as sharks and large rays wherein they behave aggressively towards each other or towards caregivers during feeding, and such behaviors can be mitigated through training [178]. Sharks at the Rotterdam Zoo were station trained using visual cues to separate species into different groups and thus reduce the incidence of chasing and biting during feeding times [179]. Similar PRT methods were used at Disney’s The Seas, where a visual indicator was used to communicate to spotted eagle rays (*Aetobatus narinari*) that food was about to be provided and thus reduced the aggressive behaviors the rays previously demonstrated towards caregivers during feeding times [178]. Rewarding targeting behaviors can be extended to apply to medical procedures, such as delivering oral medication [180].

The lack of literature focused on handling and fish welfare has historically led to a lack of information on the best practices for assessing welfare beyond injurious behaviors, with many animal welfare scientists still debating whether fish feel pain. Despite these disagreements, recent calls for more research into the well-being of fish [181] offer a positive outlook for the future of fish welfare science.

### 6.8. Invertebrates

Invertebrates includes all species that do not have a backbone, including all insects, arachnids, mollusks, crustaceans, and coral. Until recently, invertebrates in general were not considered neurologically sophisticated enough to experience suffering [182]. However, there is increasing evidence of cognitive ability in a variety of invertebrates; for instance, the honeybee (*Apis mellifera*) can learn contextual information such as patterns in a maze [183] as well as understand abstract concepts such as ‘sameness’ and ‘difference’ [184]. Such cognitive capabilities suggest that invertebrates are not only capable of experiencing their own perception of their well-being, albeit not necessarily in the same manner as vertebrates, but also possess the ability to learn to some extent. As such, it is pertinent to explore whether it is possible to provide compassionate care to invertebrates with the same level of consideration we would give to vertebrates including in the context of management and handling practices.

Traditional methods of handling invertebrates are highly variable depending on the species. For example, some species are toxic or pose other physical dangers to human handlers, necessitating the use of gloves, tongs, or other equipment to put distance between caregivers and animals [185,186]. Physical restraints such as cloth bags or nets may be used to catch and handle invertebrates; however, these methods pose a physical and psychological risk to the animal—particularly as many species of invertebrate are delicate and may be easily damaged or killed by falls. Chemical methods of restraint may also be used, such as inhalant anesthetics, although monitoring the depth of anesthesia is not easy in many cases [187].

While there is evidence that insects can learn to respond to stimuli [188], there is no peer-reviewed documented evidence of insects being trained to participate in their own care or, indeed, much discussion of what form this would take. Even without a clear understanding of the cognitive capabilities of many invertebrates in respect of their ability to learn, it is reasonable to assume that invertebrates can become habituated and desensitized to handling over time contributing to their popularity for safe use in animal–visitor interactions (AVIs). Some invertebrates, for example jumping spiders, have been demonstrated to learn and respond in response to their rearing environments, providing evidence that some species do have the capacity to remember and to learn [189]. It is evident that further exploration and research on invertebrate learning and training is necessary to understand the impacts of alternative handling methods such as using PRT on the welfare and subjective experiences of these often-overlooked taxa.

Learning in aquatic invertebrates has been variably studied by comparison [66]. In the case of cephalopods, their sensitive skin can make handling particularly stressful; however, recent guidelines suggest that cephalopods may be able to habituate to handling and PRT may reduce husbandry-related stress [190]. The ability of cephalopods, such as octopuses (*Octopus bimaculoides*) and cuttflefish (*Sepia officinalis*), to learn has been well-documented [190,191] and has contributed to the recent acknowledgement of cephalopods as sentient and thus included in many updated animal welfare regulations worldwide. Therefore, aquatic invertebrates, similar to many other species, are likely to benefit from the use of PRT for cooperation with husbandry and research procedures. Undoubtedly, invertebrates as a whole need increased focus in the welfare science literature when compared with vertebrate taxa.

## 7. The Importance of the Individual

Modern and professional handling PRT programs require an understanding of the animals as individuals and not just as members of their species. Differences may come about due to internal factors such as the sex, age, previous experiences, and personality of the individual [92], but will also be influenced by the external environment such as habitat design, group composition, and social dynamics [98]. The individual circumstances must be considered when developing handling and training plans. These diversified influences are precisely why it is impossible to devise a standardized training and handling procedure that can be used by all facilities across all members of the same species and must be considered when formulating and planning new procedures.

Individual differences in training responsiveness should also be considered, such as animals who are less bold and thus may be less willing to approach caregivers [92], or animals who are more confident and could act as ideal training partners [98]. Some animals may require more approximations before a behavior is successfully trained [95]. The personality of animals is often informally considered and incorporated into animal management decisions, such as recognizing that a more timid animal should be approached in a different, more cautious manner when compared with an animal that is confident and approaches people easily [192]. However, personality is often not systematically and programmatically included in training and handling plans, primarily due to fears of anthropomorphism and a lack of data on the personalities of individuals living in human care. There is a growing recognition that non-human primates and many other species possess personalities, and that these personalities have implications for how the animal perceives events that occur within their environments [193]. Other factors which have been demonstrated to impact cue-response and training in zoo animals include the strength of their relationship with caregivers [131].

## 8. Handling and Training in Relation to Environmental Enrichment

Environmental enrichment is defined as any additions made to the habitat or care of animals with the intention of promoting physical and psychological well-being [194]. According to some publications, human–animal interactions, including during PRT, are considered enriching [195,196,197]. For training to be maximally effective as an enrichment tool, it is important to consider how learning and enrichment schedules interact. In nature animals spend a lot of time foraging for food, sometimes across multiple attempts and spanning many hours [198]. Regarding reinforcement schedules, it is thus important to consider how often or for how long an animal could be working and engaging in different behaviors before they receive a reinforcer whether it be food, nesting materials, or another desired item [199,200]. Understanding what form reinforcement takes should focus on the value of the reward from the perspective of the animal [201,202]. As an example, trainers could utilize variable reinforcers by making it unpredictable what reward an animal is likely to receive. Preferences for reinforcement schedules and types should be considered on both a species-level and individual basis. While some individuals may demonstrate a preference for a single high-value reward in the short term, utilizing variable rewards could be beneficial for both maintaining motivation and as a form of enrichment [202].

Another area worth further research is how training can be used in conjunction with other forms of enrichment to maximize their effectiveness. A study by Fernandez et al. [203] demonstrated that training and enrichment can be combined to enhance the behavioral benefits of novel enrichment strategies for zoo-housed penguins, presenting a case study for the form that this type of training program could take in the future. As training by itself can be enriching it would be pertinent to explore in more detail the welfare implications of training as a form of enrichment, both distinct from and interconnected with all other forms of environmental enrichment provided to animals [204].

## 9. Evidence-Based Animal Handling and Training

Professional animal training programs consist of many distinct parts including carefully designed plans and processes which provide a consistent guide for all involved staff members to follow. Detailed processes and procedures that ensure caregiver time for PRT methods allow for opportunities to build positive relationships with the animals to be maximized, and ensure all procedures are a practical reflection of a contemporary animal care and well-being program. Such plans should also include time to observe the impacts of training on the behavior of animals [205] A complete checklist has been designed with respect to the discussion presented in this paper and on reflection of the many interconnecting elements of a professional and ethical animal training protocol (Table 1).

Record keeping systems range from simple Excel sheets to more advanced monitoring software such as Vigieprimates or ZIMS, including the animal care and welfare module of the latter. Predetermined rating scales or automated activity monitors should be used to document all sessions involving human–animal interactions, including animal handling and training. The focus should be not only on what is easy to record but what is meaningful [11]. Paper records are only acceptable if they are transferred to computer-based programs for review and interrogation. These records should be reviewed on a regular and—where necessary—predetermined schedule. Such records assist in the assessment of how animal adjustment to handling and training is progressing, as well as behavioral, physiological and psychological effects on the animals, and the efficacy of the process overall. Such records are essential to assessments of animal welfare in relation to handling and husbandry practices. Facilities are encouraged to conduct research projects on all species with predetermined hypotheses and questions and use the records to investigate specifics retroactively.

Animal welfare science primarily addresses individuals in terms of the whole animal. Inputs, the independent variables that impact their experience, as well as outputs, the dependent variables that tell us how animals react to inputs across time, are examined and assessed for a holistic understanding of whole animal well-being. A combination of research and other domain-relevant programs and tools should be used to assess animal welfare across time [2]. Programs should be based primarily on an outcome or animal-based measures approach such as by examining the animal’s willingness to interact [206,207], their behavior towards staff members, and whether they are demonstrating relaxed behaviors in the presence of humans. It is also necessary to look at specific animal training objectives based on the needs of the animal care program, of caregivers, and of the facility. The consistent and deliberate use of animal-based measures to assess animal welfare is new in the context of zoo animals, even if animal-based assessment has been frequently used for examining specific aspects of animal care—in particular, the effectiveness of environmental enrichment or reduction in stereotypic behavior. Practical animal welfare assessment should include outcome-based measures from the animal to provide insight into the individual’s welfare state; as an example, trainers could consider ethical measurements that gain insights into the preferences to participate or not [208].

Fairly new to zoo animal welfare approaches is the use of animal-based assessment based on the observations, knowledge, and skills of animal care staff [209]. These animal-based measures include positive and negative indicators that can be observed either directly or indirectly. Observable behavior may include play, rest, vigilance, and vocalizations [205,209,210,211,212,213], while indirect observable evidence may include the presence or absence of wounding [214], evidence of interaction with enrichment devices, or stool quality [215]. All forms of observable evidence, both direct and indirect, provide an insight into how animals are reacting to their everyday husbandry and experiences, including how they are handled and trained. However, understanding these signs requires caregivers to be experienced and knowledgeable in both recognizing and interpreting them when they are present at not only a species level, but also at an individual level. Nonetheless, subjective caregiver assessments of animal welfare based on the signs they have observed have been demonstrated to be valid, reliable, and effective tools for understanding the welfare states of the animals they care for [209,216,217,218,219,220]. Caregivers are those most familiar with the individual temperaments of the animals, and thus, are best equipped for both creating and assessing welfare plans in relation to handling and training that respect the consistent individual differences in behavior of the animals they care for [193].

## 10. Training the Trainer

An additional fundamental skill for animal care staff, beyond the important skills of promoting positive welfare and recognizing signs of well-being challenges, is to understand the science and practice of animal learning, training, and handling. While there are many different degrees, courses, and other educational opportunities available concerning the topic of animal cognition and learning, the specific job role of being an animal trainer is an unregulated profession. As such, the quality and variety of animal training skills between individuals, teams, and facilities varies greatly. There is no obligation for animal care staff to undertake additional specific training or courses to begin handling or training animals, and as such, there is a preconception that anyone who cares for animals can and should perform such activities. Consequently, some facilities may rely on outdated training and handling methods preferred by members of staff who have not received updated training on best practice in handling the species they work with in many years.

Continued professional development opportunities and education concerning animal learning and training should be considered a prerequisite for anyone working with animals in any capacity. Such training should include consideration for areas such as how to train animals living in groups, optimizing training time and outcomes, and positive human–animal interactions. A growing number of zoos have invested in employing staff specifically dedicated to developing animal behavior and training programs. The role of such animal training specialists is to be responsible for all animal training programs at the facility, as well as staying abreast of and training staff in contemporary animal handling and training methods. Modern and professional zoos should look to such cases as examples and seek to implement similar programs within their organizations to create cohesive, modern, and welfare-conscious handling and training procedures for all species. Through PRT methods and through building positive human–animal relationships founded on trust and positive emotions, the perception that caregivers have for their work and the welfare of the animals they work with will in turn improve [218,219,220]

## 11. Conclusions

We have an ethical responsibility to provide captive animals with environments that allow them to experience good well-being, including during handling and training. Animals learn all the time, whether we realize it or not, and knowledge of animal learning is fundamental to promoting optimal animal well-being. Positive reinforcement training provides opportunities to refine husbandry and care methods to best cater to the individual animal as well as group-level needs and preferences. The way we handle animals, when conducted in a manner that is positive, can improve animal well-being and the human–animal relationship as well as providing opportunities for caregivers to be proactive in their job role. All animals across different taxa in zoos and aquariums can benefit from handling and training methods which aim to create a positive experience from the animal’s perspective.

Future directions for research should examine how handling methods such as increased use of PRT can benefit a wide variety of animal species, focusing not only on more commonly studied large mammal and bird species but also reptiles, fish, invertebrates, and other taxa not currently represented in the literature. Becoming an animal care professional, including becoming an animal trainer, and maintaining the necessary knowledge of contemporary animal training should be a more regulated process. Preferably, animal training theory and practice which focus on promoting positive well-being through best practice should be incorporated into animal care and veterinarian curricula worldwide.

## Figures and Tables

**Table 1:** A checklist of the elements to consider and include when planning for ethical and evidence-based animal training and handling in a zoo and aquarium context.

Element	Description
Time	Time must be made in the daily, weekly, monthly, and yearly schedules and routines of animal care professionals for planning for training and handling, reflecting on practices, spending informal and formal time with animals to build relationships, and for conducting training sessions.
Training for all species	Training plans should be created for all species, including not only commonly trained species such as elephants and primates but also others including reptiles, fish, and birds.
Continued training for animal care staff	All animal care staff involved in the handling and training of animals, including veterinarians, should receive up-to-date continuous professional development opportunities regarding modern and ethical best practices in the handling and training of animals.
Collaboration between all involved persons	All individuals involved in the care of animals, including curators, caregivers, veterinarians, behaviorists, etc., should be involved in the development of animal training plans, and where possible all individuals should dedicate time to training to habituate animals to the presence of different people involved in their care.
Habitat design	The design of the habitat itself should be conducive to PRT, for example including specific training walls and stations where rewards can be easily administered, sleeves or windows for blood draws, and other similar design features.
Equipment	Avoid using equipment known to be aversive and coercive (such as hooks and prods) and focus on equipment that is positive from the animal’s perspective (clickers, targets, etc.).
Record keeping	Record sheets that ultimately are uploaded onto centralized digital record platforms, such as ZIMS, with consideration of the meaningful data to record regarding animal responses to training, training progress, caregiver behavior during training, etc.
Risk assessment	Training should be thoroughly planned with reflection and consideration of all potential risks to humans and animals during training and handling, including how risks will be minimized or mitigated, and contingency plans if risks occur.
Resilience and opportunities assessment	Next to a risk assessment, a resilience and opportunities assessment should be created which allows for reflection and consideration of future directions in maintaining a dynamic, creative, and innovative program supporting positive well-being and agency.
Animal choice	Training must be conducted with respect for the animal’s agency and opportunities given to not engage with handling or training if the animal chooses.
Selection of meaningful rewards and reward schedules	Rewards should be meaningful and positive from the animal’s perspective and may include food, play, access to preferred partners, and toys. The reward schedule and intervals selected must also be meaningful and positive for the animal, e.g., considering free access to food or access to food contingent on performing a correct behavior.
Trainer behavior	Individuals involved with the training of animals should be cognizant of their facial expressions, body language, tone of voice, speed of movement, and other behaviors during training and what is best suited for the species and individual that is being trained. Awareness of what is being communicated to the animal beyond giving behavioral cues is essential to building positive human–animal relationships.
Social context of training	Is the animal being trained alone, or with others? For some training animals may be separated into different areas, onto different stations, and so forth.
Reflection and evaluation	After every training session, time to reflect on what went well and what could be done better next time is key to continuous improvement.

## Data Availability

Not applicable.
